# Treatment with gemcitabine/cisplatin and durvalumab for advanced biliary tract cancer – real-world data from a multicenter German patient population

**DOI:** 10.1007/s00432-025-06239-1

**Published:** 2025-06-18

**Authors:** Florian Gerhardt, Christian Müller, Marino Venerito, Jack Chater, Raphael Mohr, Mara Egerer, Udo Lindig, Aaron Schindler, Sebastian Ebel, Janett Fischer, Maik Schwarz, Sonja Gehring, Thomas Berg, Florian van Bömmel

**Affiliations:** 1 Division of Hepatology, Department of Medicine II, Leipzig University Medical Center, Leipzig, Germany; 2 Department of Gastroenterology, Hepatology and Infectious Diseases, Otto von Guericke University Hospital, Magdeburg, Germany; 3 Medical Clinic III, Klinikum Chemnitz, Chemnitz, Germany; 4 Department of Hepatology and Gastroenterology, Charité University Medicine Berlin, Berlin, Germany; 5 Medical Clinic II, University Hospital Jena, Jena, Germany; 6 Department of Diagnostic and Interventional Radiology, Leipzig University Medical Center, Leipzig, Germany; 7 MVZ Schöneck, Schöneck, Germany; 8 Department of Gastroenterology, University Hospital Marburg, Marburg, Germany

**Keywords:** Cholangiocarcinoma, Biliary tract cancer, Liver cancer, Immune checkpoint inhibitor, Real-world data

## Abstract

**Background:**

Biliary tract cancers (BTCs) are a heterogeneous group of malignant cancers with an overall poor prognosis. For more than a decade, the standard palliative first-line therapy was cytotoxic chemotherapy with gemcitabine/cisplatin. The results of the TOPAZ-1 and KEYNOTE-966 trials have now introduced immune checkpoint inhibitors (ICIs) into first-line therapy.

**Methods:**

Between July 2022 and March 2024, we retrospectively analyzed patients with advanced BTC who were treated with gemcitabine/cisplatin and durvalumab (GCD) at collaborating German university hospitals, tertiary hospitals, and outpatient oncology practices.

**Results:**

A total of 90 patients were enrolled. The median overall survival (mOS) was 16 months, and the median progression-free survival (mPFS) was 5 months. The overall response rate (ORR) was 11.1%, and the disease control rate (DCR) was 41.1%. A perihilar primary tumor was significantly associated with better mPFS, while age group between 70 and 75 years and performance status of ECOG 2 at treatment initiation were significantly associated with poorer mOS. Adverse events (AEs) occurred in a total of 64% of patients. The most common grade 1 and grade 2 AEs included anemia (23%), thrombocytopenia (16%), neutropenia (10%), nausea (14%), and fatigue (16%). Grade 3 and grade 4 AEs included anemia (10%), thrombocytopenia (5%), and neutropenia (11%). Only one case of immune-mediated hypothyroidism (imAE) was documented.

**Conclusion:**

Our real-world data support previously reported findings and further validate ICI based therapy as the standard of care for patients with advanced BTCs.

## Introduction

Biliary tract cancer (BTC), including intrahepatic cholangiocarcinoma (iCCA), extrahepatic cholangiocarcinoma (eCCA, perihilar and distal), gallbladder cancer, and ampullary cancer, are a heterogeneous group of rare malignancies originating from the biliary tract epithelium (Valle et al. 2021). The heterogeneity extends not only to anatomical localization but also notably to molecular and biological characteristics (Valle et al. 2017; Kendre et al. 2023). BTCs are often diagnosed in advanced stages, and the prognosis is poor (Banales et al. 2020). The incidence and mortality of intrahepatic cholangiocarcinoma (iCCA) have tended to increase over the past years, while the mortality for extrahepatic cholangiocarcinoma (eCCA) has decreased in most countries (Bertuccio et al. 2019; Vithayathil and Khan 2022). However, a large proportion of patients are diagnosed in an advanced, inoperable, or metastatic stage of the disease, and only 22% can undergo surgery(Mazzaferro et al. 2020). For many years, the standard of care for palliative first-line therapy consisted of a combination therapy of gemcitabine/cisplatin (Okusaka et al. 2010; Valle et al. 2010).

The addition of immune checkpoint inhibitors (ICI) to chemotherapy has significantly improved the treatment of BTCs, and this combination treatment has recently become new standard of care (Langer; Vogel et al. 2023). Underlying mechanisms for the efficacy of ICI in BTCs are that those tumors are characterized by a chronically inflammatory and immunosuppressive peritumoral microenvironment with increased expression of immune checkpoints, such as cytokine T-lymphocyte-associated protein 4 (CTLA-4) and programmed cell death-ligand 1 (Sabbatino et al. 2016; Scott et al. 2022; Harding et al. 2023; Kawamura et al. 2023). Accordingly, the use of ICI monotherapy has shown promising results in Phase 1b and 2 studies (Piha-Paul et al. 2020). Furthermore, it is known that cytotoxic chemotherapeutics such as cisplatin and gemcitabine possess immunomodulatory properties, capable of modulating the peritumoral microenvironment by helping release tumor antigens and disrupting tumor-mediated immunosuppression (Zitvogel et al. 2013; De Biasi et al. 2014).

The results of the TOPAZ-1 study have provided evidence for the introduction of ICIs into the treatment of BTCs(Oh et al. 2022). The addition of the anti-programmed cell death-ligand 1 (anti-PD-L1) antibody durvalumab to the established first-line palliative therapy with gemcitabine/cisplatin significantly extended median overall survival (mOS) to 12.8 months (95% CI, 11.1 to 14.0) compared to 11.5 months in patients treated with gemcitabine/cisplatin (95% CI, 10.1 to 12.5). Consequently, the combination therapy of gemcitabine/cisplatin and durvalumab (GCD) is now the new standard of care in palliative first-line therapy and has been incorporated into national and international practice guidelines (Langer; Vogel et al. 2023; Alvaro et al. 2023). The results of the recently published KEYNOTE-966 study confirmed a significant survival benefit with the addition of the anti-programmed cell death-protein 1 (anti-PD-1) antibody pembrolizumab to gemcitabine/cisplatin and further strengthened the combination of ICI and cytotoxic chemotherapy for BTC (Kelley et al. 2023).

Experiences and real-world data on the treatment of patients with GCD outside of clinical trials are extremely important for daily clinical practice. So far, some real-world data have been published (Rimini et al. 2023, 2024; Olkus et al. 2024; Mitzlaff et al. 2024). Nevertheless, long-term observational data is still lacking.

The aim of this study was to investigate the clinical response and tolerability of the new standard therapy in a German patient cohort.

## Materials and methods

### Study population

We included patients diagnosed with BTC and treated with GCD at seven collaborating German university hospitals, tertiary hospitals, and outpatient oncology practices. Data were anonymized and collected retrospectively between July 2022 and March 2024.

### Treatment and monitoring

Durvalumab (1500 mg) was administered on day 1, and gemcitabine (1000 mg/m^2^)/cisplatin (25 mg/m^2^) on days 1 and 8 in a 21-day cycle. The number of therapy cycles applied varied and depended on the individual local practice and therapy recommendation of the practitioner. Subsequently, durvalumab monotherapy was possible as maintenance therapy. Prior to the official approval of the combination therapy GCD by the European Medicines Agency (EMA), the therapy was carried out following individual cost coverage by the respective health insurance of the patients concerned. Response was usually assessed after 3 months using computer tomography (CT) or magnetic resonance imaging (MRI). Clinical and paraclinical side effects were assessed at every infusion.

### Study objectives

Secondary endpoints were the investigation of adverse events (AEs) and tolerability. The primary endpoints of this study were to assess the progression-free survival (PFS), overall survival (mOS), overall response rate (ORR), and disease control rate (DCR) after initiation of GCD treatment.

### Statistical analysis

Statistical analyses were performed using IBM SPSS Statistics (version 29.0.0.0) and R (version 4.4.2). PFS was defined as the time from the beginning of GCD until disease progression, death, or loss to follow-up, whichever occurred first. OS was defined as the time from treatment initiation to the date of death. PFS and OS were reported as median values in months with an 95% confidence interval (CI). ORR was defined as the proportion of patients with complete remission (CR) and partial remission (PR) in therapy response. DCR was defined as the sum of CR, PR and stable disease (SD). Therapy response was defined by local review according to the Response Evaluation Criteria in Solid Tumors (RECIST) 1.1. AEs were defined according to the US National Cancer Institute’s Common Terminology Criteria for Adverse Events (CTCAE), Version 5.0. Immune-mediated adverse events (imAEs) were defined as adverse reactions that occurred in the context of exposure to durvalumab and were consistent with the development of an autoimmune reaction, and that were not attributable to another cause as judged by the treating physician.

## Results

### Patient baseline characteristics

Between July 2022 and March 2024, a total of 90 patients from 7 German centers were retrospectively included in the study (for baseline characteristics see Table 1). Most patients were female (57%) and under 70 years old (73%). The most common tumor etiology was iCCA (56%), followed by pCCA with 29%, gallbladder carcinoma with 13%, and dCCA with 2%. Most patients had received either prior local therapies (56%), including surgical resection and radiation, or conventional chemotherapy (14%). At the start of GCD treatment, the extent of the disease was locally advanced in 41%, metastatic in 52% and recurrent after resection in 7% of the patients. Liver cirrhosis was present in 6%, chronic hepatitis B in 2%, and chronic hepatitis C in 2% of patients, respectively. The tumor marker CA 19 − 9 was above normal values in most patients (72%) at the start of GCD treatment. Eastern Cooperative Oncology Group (ECOG) performance status at the start of GCD was 0 in 46%, 1 in 48%, and 2 in 4% of patients, respectively. Patients had received a mean of 5 cycles GCD with a range of 1–12 cycles. Median time of follow-up after treatment initiation and last observation was 5 months (range 1–32 months).

**Table 1:** Baseline characteristics of the patient cohort

Parameter (available data/number of patients)	*N* (%)
**Age group** (90/90)	
< 70 years	66 (73%)
70–75 years	15 (17%)
76–80 years	6 (7%)
> 80 years	3 (3%)
**Sex** (90/90)	
male	39 (43%)
female	51 (57%)
**Disease status** (90/90)	
locally advanced	37 (41%)
metastastic	47 (52%)
recurrence	6 (7%)
**Liver cirrhosis** (90/90)	5 (6%)
**HBV infection** (72/90)	2 (2%)
**HCV infection** (72/90)	2 (2%)
**Primary tumor type** (90/90)	
intrahepatic (iCCA)	50 (56%)
perihilar (pCCA)	28 (29%)
distal (dCCA)	2 (2%)
gallbladder carcinoma (GBC)	12 (13%)
**Previous treatment for biliary tract cancers** (90/90)	50 (56%)
**ECOG-Status** (88/90)	
0	41 (46%)
1	43 (48%)
2	4 (4%)
**Ca 19 − 9** (80/90)	
not elevated	16 (18%)
elevated	64 (71%)

### Response to GCD treatment

**Fig. 1:**
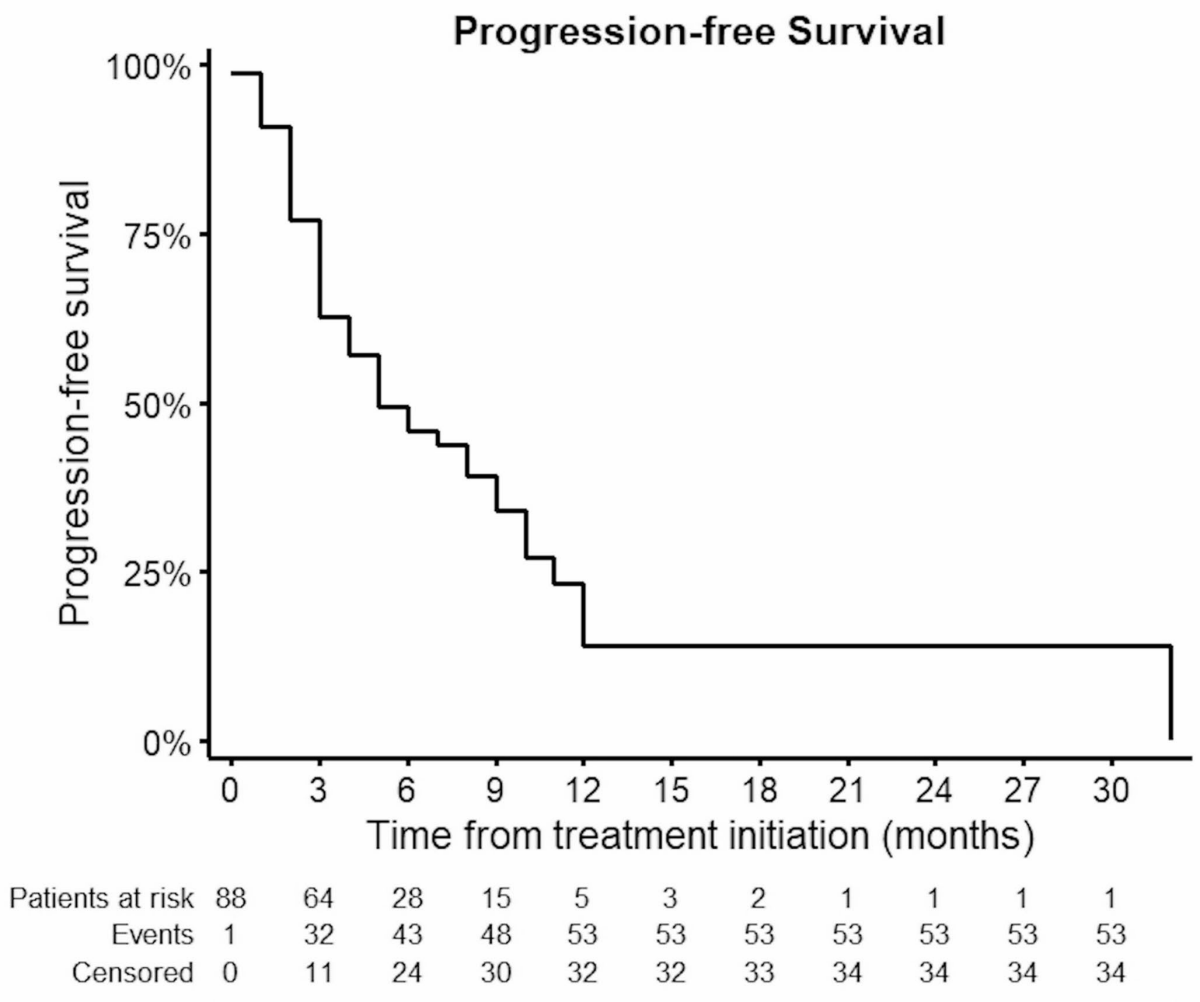
Kaplan-Meier Curve of progression-free survival (PFS) after treatment initiation of BTC with gemcitabine/cisplatin and durvalumab. BTC – biliary tract cancer

**Fig. 2:**
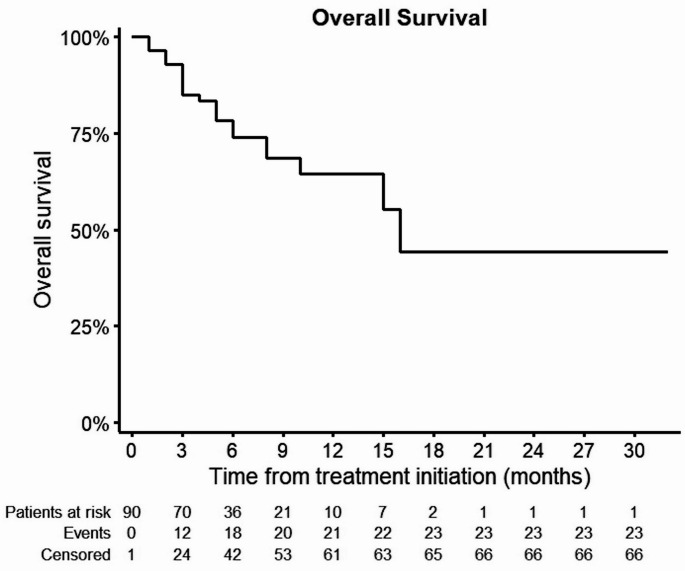
Kaplan-Meier Curve of overall survival (OS) after treatment initiation of BTC with gemcitabine/cisplatin and durvalumab. BTC – biliary tract cancer

**Fig. 3:**
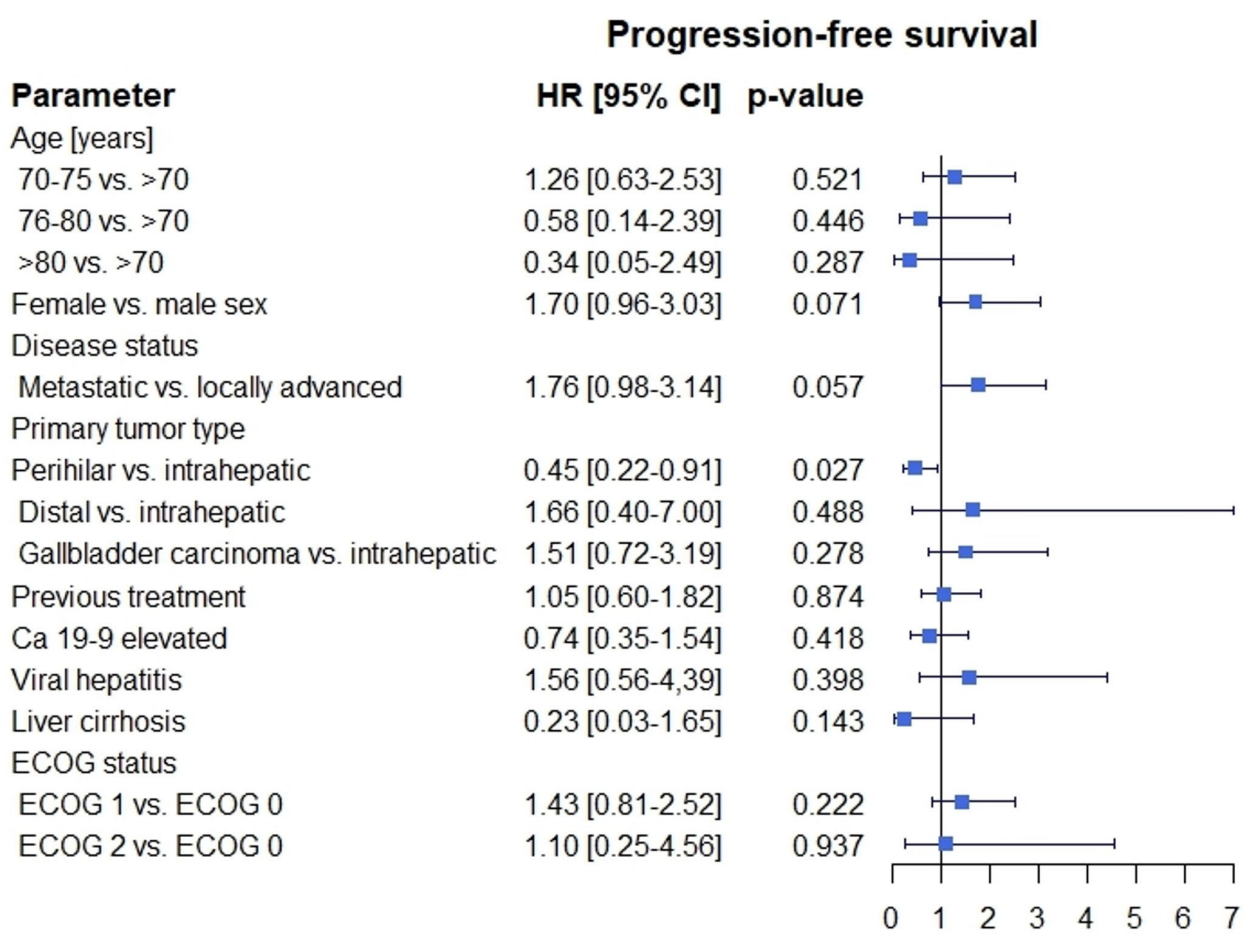
Progression-free survival in subgroups treated with GCD. GCD – gemcitabine/cisplatin and durvalumab, HR – hazard ratio, 95% CI – 95% confidence interval

**Fig. 4:**
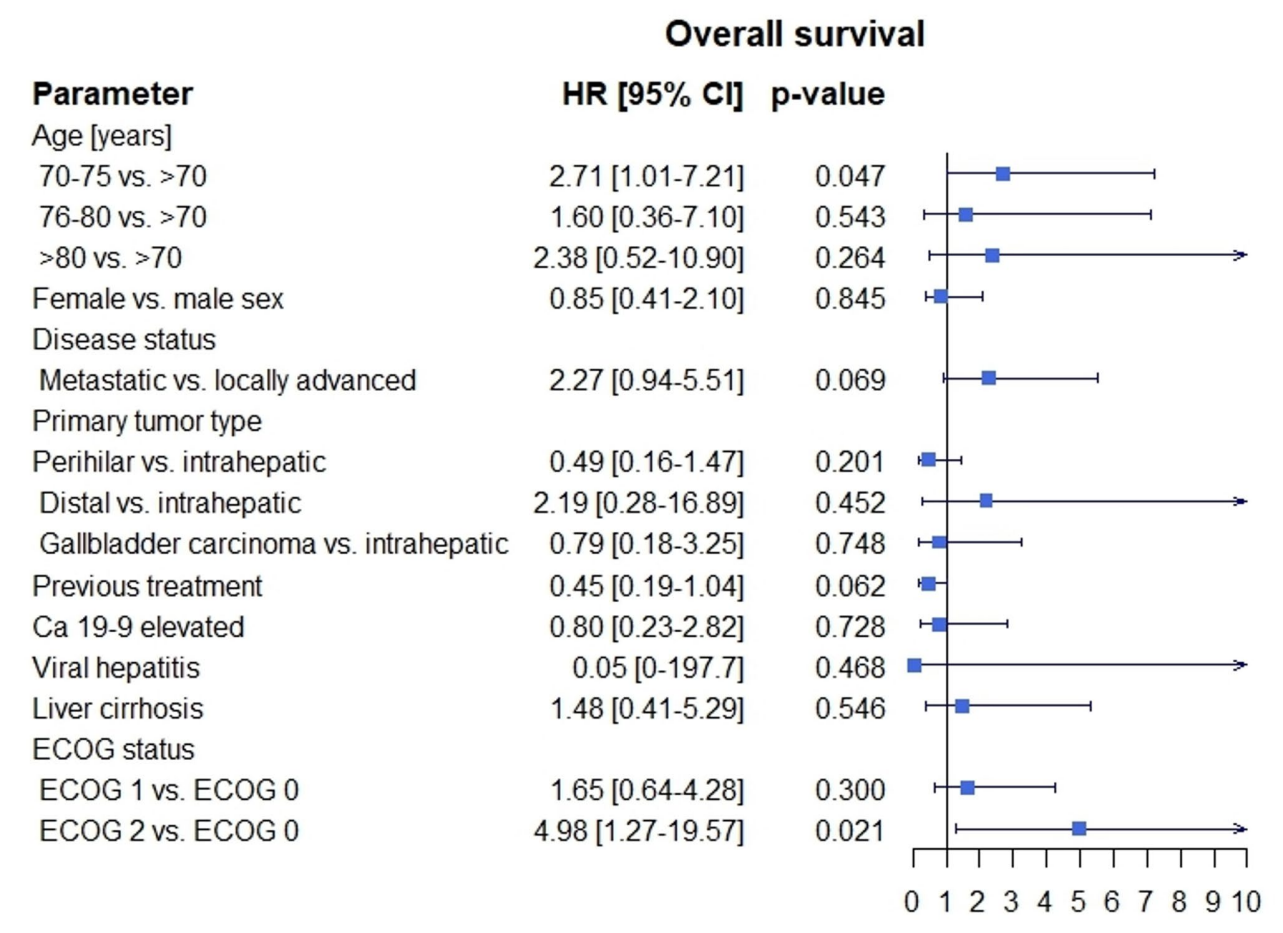
Overall survival in subgroups treated with GCD. GCD – gemcitabine/cisplatin and durvalumab, HR – hazard ratio, 95% CI – 95% confidence interval

The mPFS was 5 months [95% CI: 2.5, 7.5] (Fig. 1). The mOS was 16 months [95% CI: 13.7, 18.3] (Fig. 2). ORR was 11,1% and DCR was 41,1%, respectively (Table 2). The univariate Cox regression analysis showed that a perihilar (pCCA) primary tumor (*p* = 0.027; 95% CI: 0.22, 0.91) was significantly associated with better mPFS (Fig. 3). Regarding mOS age group between 70 and 75 years (*p* = 0.047; 95% CI: 1.01, 7.22) and performance status ECOG 2 (*p* = 0.021; 95% CI: 1.27, 19.57) at treatment initiation were significantly associated with poorer mOS (Fig. 4). Other baseline characteristics were not associated with mOS or mPFS.

**Table 2:** Summary of median progression-free survival (mPFS), median overvall ourvival (mOS), overall response rate (ORR), and disease control rate (DCR) in recently published clinical trials and real-world data. All data refer to the combination therapy of gemcitabine/cisplatin with durvalumab or pembrolizumab

	mPFS	mOS	ORR	DCR
TOPAZ-1 (Oh et al. 2022)	7.2 (95% CI: 6.7 to 7.4)	12.8 (95% CI: 11.1 to 14.0)	26.7%	85.3%
Keynote-966 (Kelley et al. 2023)	6.5 (95% CI: 5.7 to 6.9)	12.7 (95% CI: 11.5 to 13.6)	29%	75%
Rimini et al. (Rimini et al. 2023)	8.9 (95% CI: 7.4 to 11.7)	12.9 (95% CI: 10.9 to 12.9)	34.5%	87.6%
Olkus et al. (Olkus et al. 2024)	5.3 TOPAZ-1 IN 5 TOPAZ-1 OUT (HR: 0.97, 95% CI: 0.49 to 2.29)	10 TOPAZ-1 IN 10.35 TOPAZ-1 OUT (HR: 0.97, 95% CI: 0.3 to 2.6)	22.2% TOPAZ-1 IN 5.8% TOPAZ-1 OUT	61.1% TOPAZ-1 IN 58.8% TOPAZ-1 OUT
Rimini et al. (Rimini et al. 2024)	8.2 (95% CI: 7.5 to 8.9)	15.1 (95% CI: 13.4 to 29.1)	32.7%	77.8%
Mitzlaff et al. (Mitzlaff et al. 2024)	8 (95% CI: 6.8 to 9.2	14 (95% CI: 10.3 to 17.7)	28.5%	65.5%
Gerhardt et al.	5 (95% CI: 2.5 to 7.5	16 (95% CI: 13.7 to 18.3)	11.1%	41.1%

### Safety and tolerability

AEs occurred in a total of 64% of patients. Most common grade 1 and grade 2 AEs included anemia (23%), thrombocytopenia (15%), neutropenia (10%), nausea (14%), and fatigue (16%). Grade 3 and Grade 4 adverse events included anemia (10%), thrombocytopenia (5%), and neutropenia (11%) (Table 3). Remarkably, only one case of immune-mediated hypothyroidism (imAE) was documented.

**Table 3:** Summary of AEs under therapy with GCD. AEs – adverse events, imAEs - immune-mediated adverse events, GCD – gemcitabine/cisplatin and durvalumab, CTCAE - Common terminology criteria for adverse events

AE under therapy (available data/number of patients)	*n* (%)				
	CTCAE Grade 1	CTCAE Grade 2	CTCAE Grade 3	CTCAE Grade 4	CTCAE Grade 5
anemia (77/90)	13 (14%)	8 (9%)	8 (9%)	1 (1%)	
thrombocytopenia (77/90)	8 (9%)	6 (7%)	2 (2%)	3 (3%)	
neutropenia (77/90)	4 (4%)	5 (6%)	7 (8%)	3 (3%)	
diarrhea (76/90)	2 (2%)				
vomiting (76/90)	3 (3%)				
nausea (77/90)	11 (12%)	2 (2%)	1 (1%)		
fatigue (77/90)	8 (9%)	6 (9%)			
neuropathia (76/90)	4 (4%)	3 (3%)			
imAE (67/90)	1 (1%)				

### Subsequent therapy

**Fig. 5:**
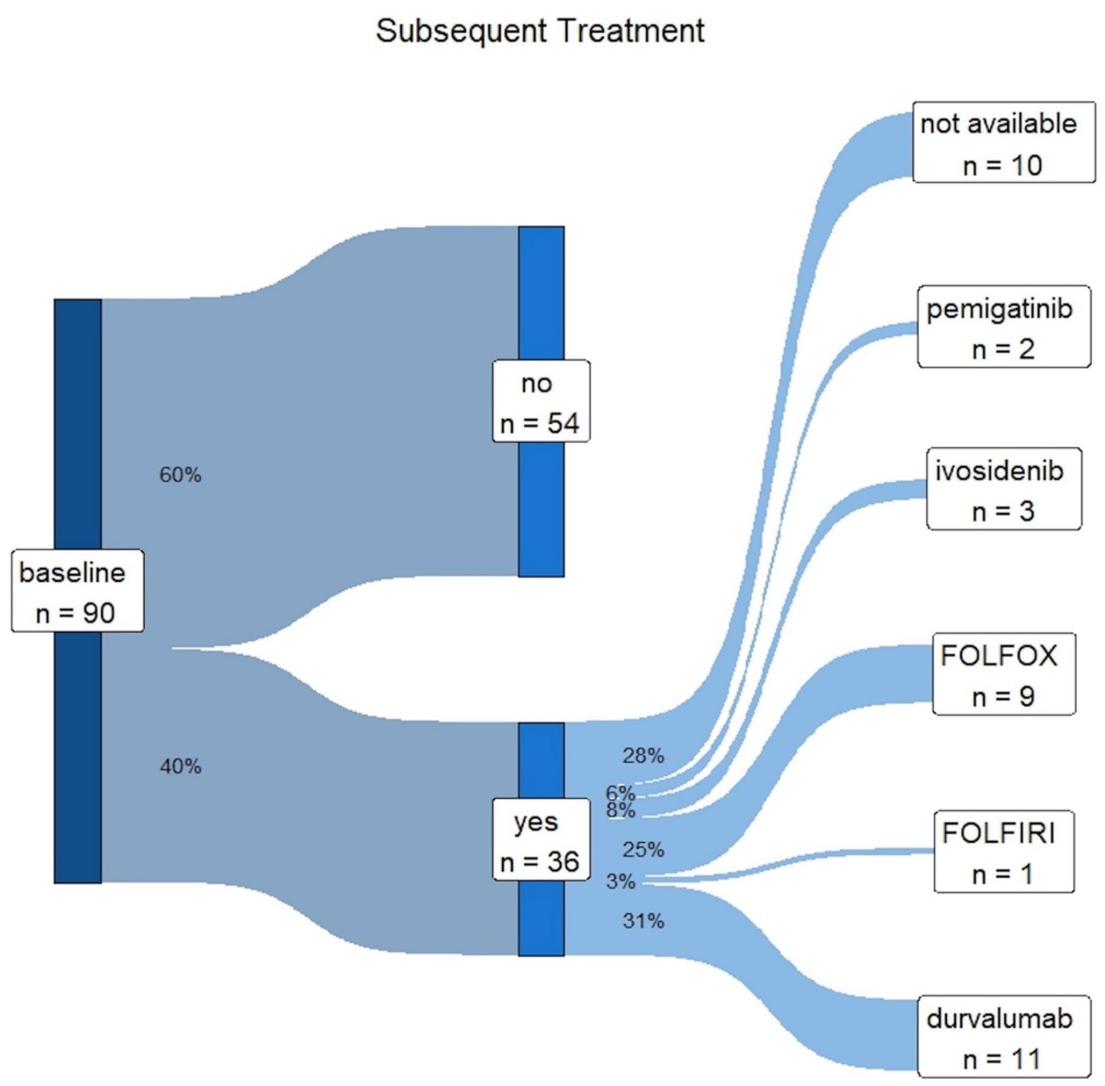
Sankey Diagram illustrating subsequent therapy for BTC. FOLFOX - folinic acid, fluorouracil, and oxaliplatin, FOLFIRI - folinic acid, fluorouracil and irinotecan

Thirty-six patients (40%) were reported to have received subsequent therapy including the isocitrate dehydrogenase 1/2 (IDH1/2) inhibitor ivosidenib (*n* = 3), the fibroblast growth factor receptor 1/2/3 (FGFR-1/2/3) inhibitor pemigatinib (*n* = 2), cytotoxic chemotherapy with FOLFOX (folinic acid, fluorouracil, and oxaliplatin) (*n* = 9) or FOLFIRI (folinic acid, fluorouracil and irinotecan) (*n* = 1), and durvalumab maintenance therapy (*n* = 11) (Fig. 5). For ten patients who received subsequent therapy, no additional information was available regarding the specific type of therapy administered.

## Discussion

The TOPAZ-1 phase III study was the first to demonstrate the clinical value of chemotherapy plus immunotherapy for previously untreated and advanced BTCs. In our real-world patient population, treatment with GCD resulted in a mPFS of 5 months and mOS of 16 months, hereby showing a favorable safety profile. Our data provide valuable information and real-world experience regarding the efficacy and tolerability of the new standard of care, GCD, for BTCs in a European patient population treated at German centers.

In the pivotal clinical trials, the median OS was extended by the addition of durvalumab to gemcitabine/cisplatin by 1.3 months (TOPAZ-1), and by the addition of pembrolizumab to gemcitabine/cisplatin by 1.8 months (KEYNOTE-966) (Oh et al. 2022; Kelley et al. 2023). However, the latest data update of the TOPAZ-1 study even shows an extension of the mOS by 1.6 months to 12.9 months without new safety signals in these highly selected patients (Oh et al. 2024). Given the palliative treatment setting and overall poor prognosis of BTCs, the extension of life is particularly significant, especially considering that the addition of durvalumab or pembrolizumab did not lead to a significant increase in Grade 3 and 4 AEs, except for immune-mediated adverse events (imAEs).

In our real-world population, the mOS was 16 months and thus notably longer as compared to the published results (Table 2). A possible explanation for the longer mOS could be the difference in pre-treatment of the patients in our population compared to the pivotal trials. Thus, more than half of the patients in our study had received surgical or loco-regional treatments prior to systemic treatment with GCD. In contrast, in the TOPAZ-1 study, only patients whose surgical therapy or adjuvant therapy (chemotherapy and/or radiation) had been completed more than 6 months in the past could be included (Oh et al. 2022). Similarly, in the KEYNOTE-966 study, only patients who had completed adjuvant or neoadjuvant therapy 6 months before the diagnosis of unresectable or metastatic cholangiocarcinoma were included (Kelley et al. 2023). Owing to the real-world character of our study, decisions regarding previous treatment and time point of initiation of GCD in our cohort varied greatly, depended on local practices and individual expertise. A plateau in the Kaplan-Meier survival curve for mOS is also notable. We attribute this plateau primarily to patients who received subsequent therapy after disease progression under GCD. To avoid losing valuable time in the event of disease progression before initiating a subsequent therapy, a detailed molecular-pathological tumor characterization should ideally be performed at the time of initial diagnosis. Nevertheless, it must be noted that in this study, only very limited data on molecular-pathological tumor characterization were available, as no standardized testing was conducted and, consequently, such data were not collected. In contrast, the mPFS of 5 months in our cohort was shorter compared to other published data. The shortened mPFS could, among other factors, may be attributed to the sample size, the retrospective multicenter study design with no predefined patient selection, and the lack of standardized response assessments (i.e., response assessment modalities and timing).

Treatment with GCD was safe and well tolerated in most patients, which aligns with the results from the TOPAZ-1 and KEYNOTE-966 trials (Oh et al. 2022; Kelley et al. 2023). Most documented AEs were attributable to cytotoxic chemotherapy, primarily involving bone marrow toxicities and general symptoms like fatigue and nausea (Table 2). A limiting factor to note, however, is that the number of reported AEs is lower compared to previously published data. In the TOPAZ-1 study, 75.7% Grade 3–4 AEs were reported, while in the Keynote-966 study, 14% Grade 1–2 AEs and 79% Grade 3–4 AEs were reported.

Moreover, the addition of durvalumab did not lead to a significant increase in imAEs in our population. In the TOPAZ-1 study, 12.7% of imAEs were reported for the combination therapy GCD, with the most frequent immune-related events being hypothyroidism, dermatitis/rash, hepatic issues, and adrenal insufficiency (Oh et al. 2022). In the KEYNOTE-966 study, 22% of imAEs were associated with the combination therapy of gemcitabine/cisplatin and pembrolizumab (Kelley et al. 2023). Interestingly, only one patient in our population had showed an imAE which was immune-mediated hypothyroidism. A possible reason for the low number of documented imAEs in our study could be due to under reporting. However, together with the encouraging response data, the rare finding of imAEs as well as of AEs grade 3 and 4 supports the concept of combining ICI and cytotoxic chemotherapy in BTC patients.

It needs to be pointed out that all adverse events were analyzed retrospectively and were primarily derived from laboratory findings or the respective patient documentation systems of the participating centers. Furthermore, it must be acknowledged that, unlike laboratory parameters, AEs in form of general symptoms are often not systematically assessed or documented. This may account for the incomplete, imprecise, or missing data. However, it also reflects the clinical real-world setting beyond controlled clinical trials.

Other results from real-world BTC patients undergoing treatment with GCD have recently been published (Rimini et al. 2023, 2024; Olkus et al. 2024; Mitzlaff et al. 2024) (Table 2). Rimini et al. reported results from the largest real-world cohort to date (Rimini et al. 2024). Reported mPFS, and mOS were 8.2 (95% CI: 7.5 to 8.9) and 15.1 (95% CI: 13.4 to 29.1). ORR was 32.6% and the DCR was 77.8%. Any grade AEs occurred in 92.9% and imAEs occurred in 20% of patients.

In summary of all real-world data published to date, it must be concluded that therapy with GCD represents an effective and safe treatment even beyond clinical studies with a highly selected patient population. Therefore, therapy with GCD should undoubtedly be applied as a first-line therapy. Given that most of the patients in our analysis would not have met the TOPAZ-1 or KEYNOTE-966 eligibility criteria primarily due to pretreatment, in conjunction with our results, it can be assumed that many patients with advanced BTC, reduced performance status, and pretreatments could still benefit from therapy with GCD.

One of the strengths of our study is the extended observation period for many patients, along with the involvement of experienced centres and practitioners. Another notable strength is the relatively high number of patients included. In addition, tolerability and efficacy were confirmed in a European patient cohort, whereas most patients recruited for the registration trails were Asian. This is particularly important given the rarity of BTCs in Europe compared to Asian countries, where the incidence of BTCs is higher (Rumgay et al. 2022). A limitation of our study is the retrospective design with no predefined protocol for the systematic and standardized recording and documentation of AEs, for response assessment, response assessment modalities, or timing of assessment, which were left to the discretion of the local physician. Nevertheless, the consistency between the complete case data and the imputed data speaks for the robustness of our results. However, the possibility of unrecognized distortions due to missing data cannot be completely ruled out.

In conclusion, our real-world data support previously reported findings and further validate GCD as the standard of care for patients with advanced BTCs. Treatment with GCD was effective and well tolerated as part of a multimodal treatment with prior surgical or loco-regional treatment and subsequent follow-up therapy. Further trials with patients beyond the inclusion criteria of the pivotal trials are needed to define the range of patients who will benefit from the addition of ICI to gemcitabine and cisplatin. Moreover, it seems time to develop strategies for the management of BTC patients who have a long-term response to GCD to maximize the benefits of treatment. This can include local, surgical, or systemic treatments, depending on the individual course.

## Data Availability

No datasets were generated or analysed during the current study.
